# Analysis of maintaining human maximal voluntary contraction control strategies through the power grip task in isometric contraction

**DOI:** 10.1038/s41598-023-51096-y

**Published:** 2024-01-12

**Authors:** Jinyeol Yoo, Woong Choi, Jaehyo Kim

**Affiliations:** 1 Unmanned/Intelligent Robotic Systems, LIG Nex1, 338, Pangyo-ro, Bundang-gu, Seongnam-si, Gyeonggi-do Republic of Korea; 2 College of ICT Construction and Welfare Convergence, Kangnam University, 40, Gangnam-ro, Giheung-gu, Yongin-si, Gyeonggi-do Republic of Korea; 3 Department of Advanced Convergence, Human Ecology and Technology, Handong Global University, 558, Handong-ro, Buk-gu, Pohang, 37554 Republic of Korea

**Keywords:** Motor control, Sensorimotor processing

## Abstract

Power grip force is used as a representative indicator of the ability of the human neuromuscular system. However, people maintain the power grip force via different control strategies depending on the visual feedback that shows the magnitude of the force, the magnitude of the target grip force, and external disturbance. In this study, we investigated the control strategy of maintaining the power grip force in an isometric contraction depending on these conditions by expressing the power grip force as a person’s Maximal Voluntary Contraction (MVC). The participants were asked to maintain the MVC for each condition. Experimental results showed that humans typically control their MVC constant abilities based on proprioception, and maintaining the target MVC becomes relatively difficult as the magnitude of the target MVC increases. In addition, through interactions between the external disturbance and the target MVC, the MVC error increases when the target MVC increases and an external disturbance is applied. When the MVC error reaches a certain level, the offset effect is expressed through visual feedback, helping to reduce the MVC error and maintain it smoothly, revealing a person’s MVC maintenance control strategy for each condition.

## Introduction

Humans use their hands daily to interact with the environment and control several arm muscle movements^1^. The control of human muscle movements is achieved through the cooperation of the nervous system, such as the spinal cord, cerebellum, and primary motor cortex^2^. The interaction between motor mechanisms created by the cooperation of these neural systems and sensory mechanisms related to the explorative function of the fingers enables the performance of refined motor behaviors, such as grasping an object^3^. Humans use power grip motions, which is a form of grasping ball or cylindrical shaped objects, and pinch grip motions, which is a form of gripping with only the thumb and index finger, to hold objects and perform tasks according to their size, shape, and progress through the object^4,5^. Among these, the power grip force, which is the force produced by a person’s power grip, is a dominant parameter representing the ability of the human neuromuscular system^6–9^. So, power grip force is widely used in bioengineering research and rehabilitation fields. Previous studies have shown that the ability of the neuromuscular system to generate maximum power grip force declines with age. These studies have compared the differences in ability between the general population and stroke patients^6,8,10,11^. In addition, in the isometric contraction position, where the joint angle and muscle length do not change during muscle contraction, the task of maintaining power grip force is performed by the activity of the primary motor cortex^2,12,13^. However, according to previous studies, even if the same power grip task is performed, the primary motor cortex area and degree of activation within the brain differ depending on the visual feedback showing the current state or magnitude of the force, and external force^14–17^. The findings of these studies imply that each condition’s primarily used motor sensory function differs^18,19^. In other words, even if the same power grip task is given, each goal is accomplished using a different control strategy depending on the given conditions.

The goal of the current study was therefore to find the parameters that affect the power grip task more dominantly. To this end, we assumed that even if participant proceeds with the same power maintenance task, setting the visual feedback, target power grip force and external disturbance would lead to different results. For this purpose, data normalization was performed, and the target power grip force was expressed as the Maximal Voluntary Contraction (MVC), which is the maximal force-generating capacity of a muscle or a group of muscles in humans^20^.

This study investigated a force maintenance control strategy to maintain power grip force under various conditions in an isometric contraction posture. Experiments were conducted on 25 participants under conditions for providing visual feedback (VFB), the magnitude of the Maximal Voluntary Contraction (target MVC) to be maintained, and external disturbance conditions (Disturbance Level) to study human power maintenance control ability. The MVC error was calculated for each condition. Statistically, significant differences were observed when analyzing the sensory feedback and control strategies used by humans to maintain a constant power grip force.

**Figure 1:**
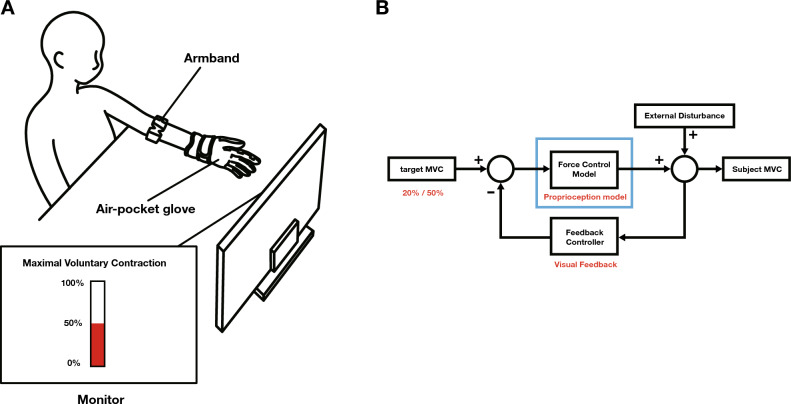
Experimental overview*.* (**A**) Participants are made to wear an armband and an air-pocket glove. As shown in Table 1, we divided the experimental task cases into four cases according to the conditions given to the participants. In the case of C1 and C3, which provide visual feedback, participants can check their MVC in real-time through the monitor. In addition, by putting air into the air-pocket glove worn by the participant to open the hand, we provided an external disturbance that hinders the isometric contraction task maintained in the power grip posture. (**B**) A block diagram showing the MVC maintenance mechanism. Participants use a proprioception model to main the target MVC. At this time, an external disturbance is applied to the participant, and the participant uses visual feedback to match the Participant’s MVC as much as possible with the target MVC.

Before experiment, we predicted that the MVC error would be large when no visual feedback was provided, when the target MVC was large, and when external disturbances were present. The first reason for making that prediction was because people perform many activities through visual feedback throughout their lives, so we thought that it would be difficult to maintain MVC if visual feedback disappeared. Second, when a person produces a relatively large force, more energy is needed than when a person produces a relatively small force, so more energy is needed to maintain and control it. Therefore, we thought that it would be difficult to maintain MVC. Also, when the environment changes, people spend a lot of time and energy adapting to it, but when a disturbance occurs, more energy is spent adapting to the variable, so we thought that it would be difficult to maintain MVC as a result. Based on these predictions, we developed seven two-tailed null hypotheses: (1) The visual feedback significantly did not affect MVC error, (2) the target MVC significantly did not affect MVC error, (3) the disturbance level significantly did not affect MVC error, (4) the interaction between the visual feedback and the target MVC significantly did not affect MVC error, (5) the interaction between the visual feedback and the disturbance level significantly did not affect MVC error, (6) the interaction between the target MVC and the disturbance level significantly did not affect MVC error, and (7) the interaction between the visual feedback, the target MVC and the disturbance level significantly did not affect MVC error. An evaluation experiment for the participants is shown in Fig. 1.

## Results

As a result of statistical analysis, it was confirmed that the target MVC ($F \left(1, 24\right)=51.492, p=0.000,$ partial ${\eta }^{2}=0.682$, item A in Table S1), the Disturbance Level ($F \left(1.582, 37.958\right)=25.203, p=0.000,$ partial ${\eta }^{2}=0.512$, item A in Table S1), the interaction between the visual feedback and the target MVC ($F \left(1, 24\right)=15.685, p=0.001,$ partial ${\eta }^{2}=0.395$, item A in Table S1), the interaction between the visual feedback, the target MVC, and the Disturbance Level ($F \left(1.536, 36.866\right)=9.981, p=0.001,$ partial ${\eta }^{2}=0.294$, item A in Table S1) significantly affected the MVC error. Through the results, we confirmed that among the seven null hypotheses, only the first, fifth, and sixth null hypotheses satisfy the hypothesis.

**Figure 2:**
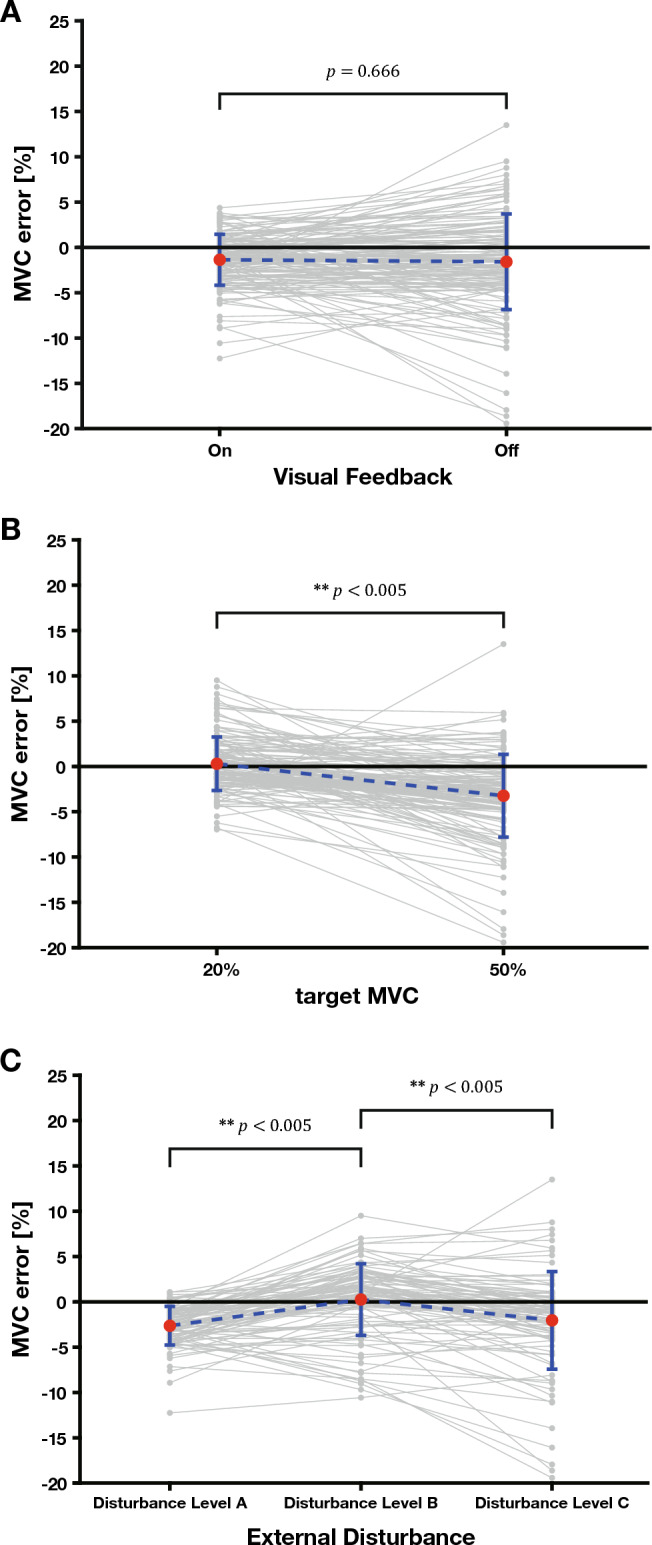
Comparison of the average MVC error for each condition. (**A**) Comparison graph of average MVC error according to presence or absence of visual feedback (*p* value = 0.666). (**B**) Comparison graph of average MVC error according to the target MVC (*p* value = 0.000). (**C**) Comparison graph of average MVC error according to External Disturbances (*p* value between Disturbance Level A and B = 0.000, *p* value between Disturbance Level A and C = 0.796, *p* value between Disturbance Level B and C = 0.000).

Figure 2 shows the statistical results of comparing MVC errors according to visual feedback, target MVC, and Disturbance Level. We can see from Fig. 2A that there is no statistically significant difference in MVC error between the case $(-1.360 \pm 4.082)$ that provided visual feedback to the participant and the case $(-1.587 \pm 4.387)$ that did not provide visual feedback (item B in Table S1). In other words, visual feedback did not significantly help the participant maintain the target MVC.

Figure 2B shows that the MVC error differs significantly depending on the target MVC (item C in Table S1). When the target MVC is 20% $(0.292 \pm 2.847)$, the error is smaller compared to when it is 50% $(-3.239 \pm 5.623)$, indicating that it is easy for the participant to maintain the MVC if the target MVC is small. This finding aligns with previous studies proving that when the target MVC is increased, the magnitude of the MVC that the participant’s muscles must produce increases, and the noise of the muscles increases, resulting in a greater error^21^.

In addition, Fig. 2C shows that the MVC errors in Disturbance Level A $(-2.639 \pm 3.711)$ and Disturbance Level C $(-2.040 \pm 3.652)$, in which the muscle lengths are stationary, do not differ significantly. Also, in Disturbance Level B $(0.259 \pm 5.341)$, where the muscle length changes because of external disturbances, the MVC error differs significantly from other Disturbance Levels (item D in Table S1). In other words, when the participant’s muscle length increased and the joint moved because of external disturbances, there was a difference in the ability to maintain the MVC.

**Figure 3:**
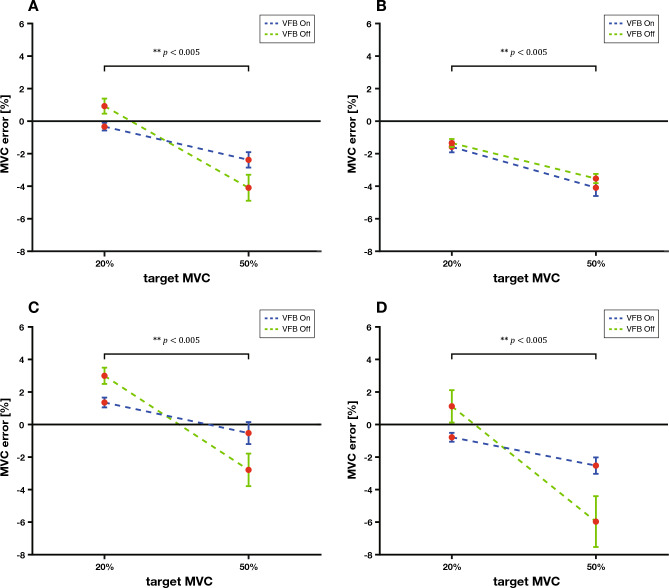
Average MVC error graph according to the interaction of each condition. (**A**) MVC error graph according to the interaction between visual feedback and the target MVC (*p* value = 0.001). (**B**) MVC error graph according to the interaction between visual feedback, the target MVC, and the External Disturbance in Disturbance Level A (*p *value = 0.001). (**C**) MVC error graph according to the interaction between visual feedback, the target MVC, and the External Disturbance in Disturbance Level B (*p* value = 0.001). (**D**) MVC error graph according to the interaction between visual feedback, the target MVC, and the External Disturbance in Disturbance Level C (*p* value = 0.001).

Figure 3A presents the statistical results of comparing the MVC errors for the conditions in which the visual feedback and the target MVC interacted. First, through Fig. 2A, we confirmed that visual feedback does not help significantly in maintaining the MVC. Figure 3B confirms that the MVC error increases when the target MVC increases. However, Fig. 3A shows that when visual feedback was not provided, the magnitude of the MVC error, which increased with increasing target MVC, was much larger than when visual feedback was provided.

Figure 3B–D presents the statistical results of comparing MVC errors for the conditions in which the visual feedback, target MVC, and external disturbances interacted. Thus, it can be confirmed that the results illustrated in Fig. 3A are not similarly applied to all Disturbance Levels but only to Disturbance Levels B and C, where an external disturbance exists. In contrast, at Disturbance Level A, external disturbance exists. The magnitude of the increasing error is similar when the target MVC increases from 20 to 50%, regardless of the visual feedback.

Additionally, although the subjects looked at the actual value of MVC error and controlled to reduce it, we conducted a three-way repeated measures ANOVA test on the absolute MVC error for each condition (S2 Table). What is noteworthy in that results is that the visual feedback ($F \left(1, 24\right)=62.443, p=0.000,$ partial ${\eta }^{2}=0.722$, item A in S2 Table) significantly affected the absolute MVC error.

## Discussion

In this study, we analyzed MVC maintenance control strategies in humans using MVC maintenance experiments. Evaluation experiments were conducted under visual feedback, target MVC, and external disturbance conditions. Based on the experimental results, the following sections will be discovered: (1) Human MVC maintenance ability and the offset effect of visual feedback that complements the limitations of that ability, (2) analyzing human MVC maintenance control strategies, and (3) applications and future work.

### Human MVC maintenance ability and the offset effect of visual feedback complement the limitations of that ability

This experiment confirmed that visual feedback does not significantly help participants maintain their MVC bias (Fig. 2A). However, as a result of the experimental data for each condition, the overall error did not deviate significantly, and most errors were close to zero. In other words, there is a dominant parameter that helps participants maintain their MVC. There were two types of feedback available to the participants in this experiment: visual feedback provided by the evaluation system and proprioception feedback the participants provided by themselves. However, because visual feedback does not significantly help maintain MVC, the participants’ MVC maintenance control ability-based proprioception helps them maintain MVC bias properly^1,22^. In other words, sufficient maintenance effects can be achieved using human MVC maintenance capabilities without additional visual feedback. These findings confirm the results of previous studies that the human body does not use the senses more than necessary to control them efficiently without consuming energy^23^.

However, as the target MVC increases, the MVC error increases, confirming that the human MVC maintenance ability is limited according to the target MVC (Fig. 2B)^1^. We found that this helps to reduce the MVC error under conditions in which visual feedback and target MVC interact to compensate for this limitation (Fig. 3A). Although visual feedback does not help maintain the MVC, we find the ‘offset effect’ that compensates for the limitations of the MVC maintenance ability, increasing the MVC error as the target MVC increases. However, only Disturbance Levels B and C with external disturbances showed an offset effect (Fig. 3C–D). In contrast, Disturbance Level A without external disturbances did not show an offset effect (Fig. 3B). In other words, visual feedback can obtain an offset effect that reduces the increase in error if an external disturbance and interaction are applied with a large force.

On the other hand, we performed statistical analysis on the absolute value of MVC error in the same way. The results of statistical analysis showed that visual feedback does not reduce the bias of MVC error but has an effect on reducing variation. In this study, only the signed MVC error was shown to the subjects and the experiment was conducted using this as a factor. However, if we design and analyze an experiment with absolute MVC error as a factor, it is believed that we will be able to find out more about people’s MVC maintenance strategies.

### Analyzing human MVC maintenance control strategies

From the results of this experiment, we confirmed that there is a difference in MVC maintenance when an external disturbance moves the finger joint and that the length of the muscle that moves the finger joint changes. The MVC errors were not statistically significant at disturbance levels A and C, where the muscle length was stationary. At Disturbance Level B, where the muscle length changed, the MVC errors differed significantly from the other Disturbance Levels (Fig. 2C).

An external disturbance was generated from disturbances A to B, and as the magnitude increased, the muscle length increased. As a result, the MVC error increases, and the offset effect provided by the visual feedback acts. However, the statistical difference in MVC errors between Disturbance Levels A and B indicated that the participants did not fully obtain the offset effect through visual feedback at Disturbance Level B. In other words, the offset effect was not applied simultaneously with the external disturbance. However, the offset effect compensates for the increase in MVC error when it reaches a certain threshold level. Although the visual feedback provides an offset effect, the effect is not expressed from the beginning of Disturbance Level B. Therefore, it is reasonable that the MVC errors at Disturbance Levels A and B are statistically significant.

The muscle length was maximized and maintained from Disturbance Levels B to C. Because Disturbance Level C has different muscle lengths from Disturbance Levels A and B, the MVC error should also differ significantly from the errors in Disturbance Levels A and B. However, it can be interpreted that the offset effect was sufficiently expressed, and the error changed, as shown in Disturbance Level A. That is, although the error in Disturbance Level B is closer to zero than those in Disturbance Levels A and C, the offset effect changes the error in Disturbance Level C to one similar to that in Disturbance Level A, indicating that maintaining a constant MVC difference of bias is a much smoother maintenance strategy used by humans than trying to accurately zero when an external disturbance is applied.

Through this study, it was revealed that there are a total of three strategies for controlling humans to maintain MVC according to external disturbance. First, human controls generally maintain MVC using their inherent MVC maintenance ability. Secondly, as the magnitude of the target MVC to be maintained increases, it becomes relatively difficult to maintain the MVC, and the MVC error increases. Third, if the MVC error increases and reaches a certain level, the offset effect is expressed through visual feedback, which helps to reduce the MVC error and maintain it smoothly.

### Applications and future work

This study investigated strategies for controlling humans to maintain the MVC according to external disturbances. The results of this study can be applied to tasks that maintain MVC. They can be used effectively in the fields of rehabilitation and robot collaboration.

In previous studies, the power grip force during an isometric contraction task was used as an indicator of the neuromuscular ability of stroke patients. As a result of this study, the interaction between visual feedback and target MVC was found to affect the maintenance of the MVC^6,8,10,11^. Therefore, when rehabilitation is performed with isometric contractions, it will have a greater effect if performed while visually showing the magnitude of a patient’s strength.

In addition, they can be applied to collaborative fields, such as humans and robots, by applying force to each other and lifting and transporting objects together. In most existing environments where humans and robots collaborate, collaborations are carried out by providing the location and speed of robots working together with people. However, it can be much more helpful if it provides a human with a robot’s position and a speed indicator and visually provides the magnitude of its force as a new indicator.

In this study, an external disturbance was applied to the participant by placing air in an air-pocket glove, and the person’s control strategies to maintain the MVC according to the external disturbance were studied. In previous studies applying external forces to humans, the quantitative external force value was measured, and the studies were conducted^24,25^. However, in this study, the magnitude of the external disturbance caused by the air-pocket glove was not calculated quantitatively. Additionally, the magnitude of the participant’s force was expressed as an MVC percentage and not as an absolute value. In future work, other quantitative results will be obtained if the experiment is conducted by measuring the magnitude of the external force and the participant’s force, which can be obtained to support Hill’s muscle model, which represents the length and force of the muscles as spring and contractile components^26^.

## Materials and methods

### Participant

An experiment was conducted on 25 right-handed university students aged $22.96 \pm 2.07$ years who did not have disabilities in the upper limb movement. We explained the experimental plan and purpose to all participants in advance. Informed consent is obtained from all participants, and we conducted the experiment in compliance with appropriate regulations and guidelines. The experiment was conducted with approval from the Handong Global University Institutional Review Board (protocol code: 2022-HGUA009; date of approval: 08 June 2022).

### Experimental system

The participants were instructed to make a power grip by making a fist with their left hand and to perform an isometric contraction task while maintaining the power grip force at a constant magnitude. Based on the fact that the muscles that move the fingers are inside the forearm muscles, the surface electromyogram (sEMG) of the forearm muscles was measured, and the sEMG signal was then converted into the MVC percentage of the power grip force^9,27–29^. An armband (Myo, Thalmic Labs) that measures sEMG signals was worn on the forearm muscle area located 2 cm from the left elbow to the wrist, and an air-pocket glove (HiiiiiFive, HiiiiiFive®) that provided external disturbance to the fingers was worn on the left hand^30–32^. During the experiment, the magnitude of the participant’s MVC was provided as a bar graph on the monitor to be checked in real-time^33^.

### Experimental design

As mentioned in previous studies, people achieve their goals with different control strategies depending on visual feedback, target MVC, and external disturbances; therefore, the experiment was conducted under three conditions^15–17^. Under these three conditions, the core goal of the experiment was to give participants a task to maintain MVC and to determine the control strategies they use to reduce MVC errors that occur during the task.

First, the task cases were divided according to the presence or absence of visual feedback and the target MVC (Table 1). When visual feedback was provided, the participant was allowed to maintain the feedback while viewing it in real-time. When visual feedback was not provided, the participant maintained the target MVC. If the participants maintained the target MVC well through visual feedback, they were instructed to continue maintaining it with the visual feedback removed.

**Table 1:** Task cases of participants according to conditions.

Case	Visual feedback	Target MVC (%)
C1	*On*	20
C2	*Off*	20
C3	*On*	50
C4	*Off*	50

**Figure 4:**
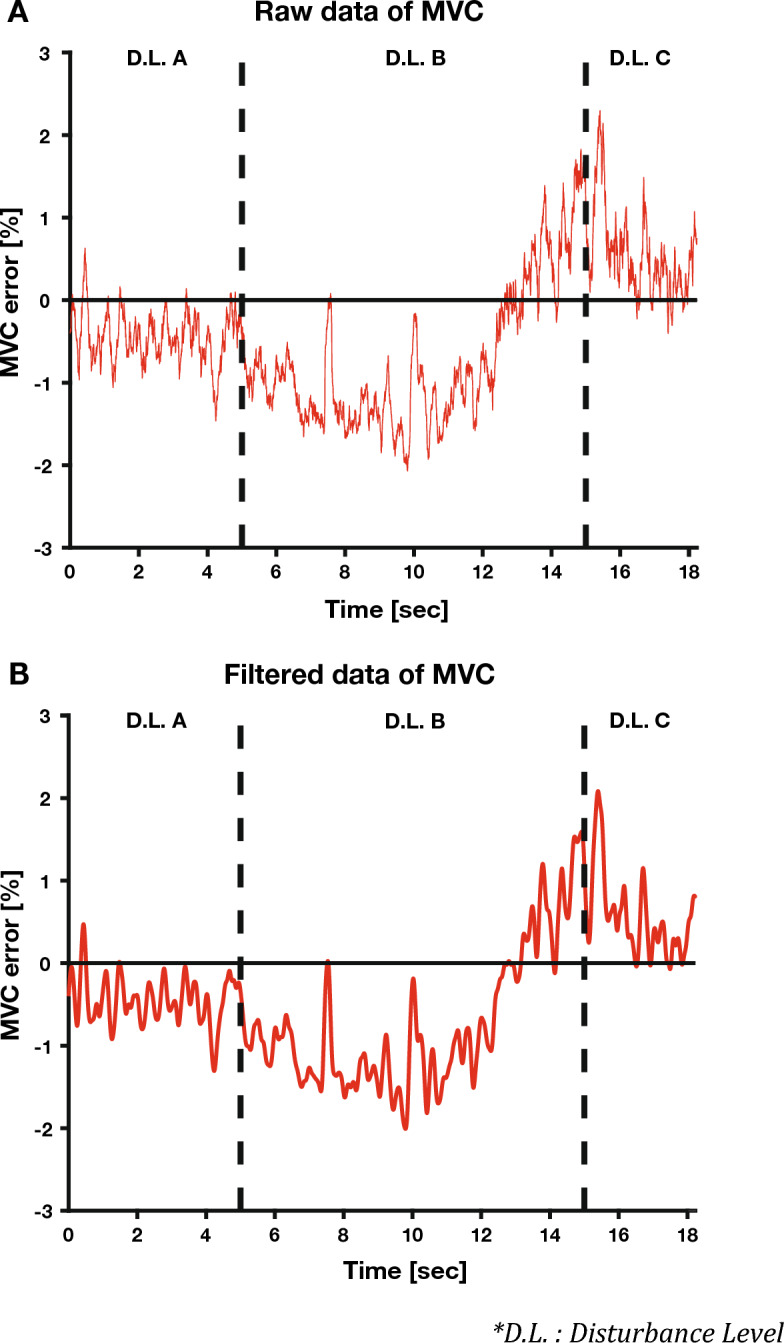
MVC error graph at the C1 task of representative participants. It is divided into Disturbance Level A sections where no external disturbance is applied, Disturbance Level B sections where a gradually increasing external disturbance is applied, and Disturbance Level C sections where the external disturbance is maximum. (**A**) MVC raw data before filtering. (**B**) MVC filtered data after filtering. It can be confirmed that the sensor and muscle noise are greatly improved using the 3rd Butterworth filter.

Additionally, each case was classified according to when the external disturbance was applied. In other words, each case was divided into Disturbance Level A when the disturbance was not applied, Disturbance Level B when the internal pressure of the air-pocket glove was linearly increased by 0.085 bar per second, and Disturbance Level C when the internal pressure of the air-pocket glove was maximized (Fig. 4). The disturbance conditions were always fixed in the order of disturbances A, B, and C, and disturbance levels A, B, and C lasted 5, 10, and 3 s, respectively. The participants experimented three times for each case, and a 2-min break was provided for each experimental trial. In addition, prior practice time was provided to maintain the MVC sufficiently before the experiment so that the participants could familiarize themselves with the system.

### Data processing

We measured the sEMG in all directions of the forearm muscle using armbands and added it to express it as MVC for data normalization. Before that, the noise of the sEMG signal was signal-dependent and increased linearly with the absolute value of the signal^34^. A quasi-tension filter, a type of 2nd low-pass filter, was used to remove high-frequency noise from the sEMG signals of the participant^34,35^. The transfer function of the quasi-tension filter used in the experiment is expressed by Eq. (1).

1 $$Quasi \;tension \;filter=\frac{36.84}{{s}^{2}+27.32s+178.4}$$

The mean absolute error (MAE) was used to convert the sEMG signal into the MVC percentage during filtering^36,37^. After setting the sEMG values when the participant applied maximum force in the isometric contraction posture and when the participant did not apply any force at all to reference values of 100% and 0%, respectively, the participant’s sEMG signal was represented by the MVC of the participant in real-time using the MAE (Eq. 2 and Fig. 4A).

2 $$Total \;Contraction \;Level[\%]=\frac{100}{Standar{d}_{100\%}-Standar{d}_{0\%}}(sEMG-Standar{d}_{0\%})$$

Figure 4A shows that armband sensor noise and muscle noise were mixed in the participant’s MVC signal. The MVC signal’s power spectral density (PSD) was calculated using Welch’s method with a positive FFT to determine the appropriate cut-off frequency of the low-pass filter^38^. Welch’s method divides data in a long-time area into sections and averages the PSDs obtained by the Fourier Transformation (FT) of the data in each section. PSD bias occurs because the length of the FT signal is shortened, and the frequency resolution decreases. However, the averaging operation removes randomness to reduce the PSD deviation^39,40^. Welch’s method was used to remove sensor and muscle noise from the PSD and to emphasize the components containing the participant’s movement intention. The window size used in Welch’s method was approximately 10 s, half the signal length. The FT was performed by moving a 10-s window at 1-s intervals, and the resulting PSD was averaged.

**Figure 5:**
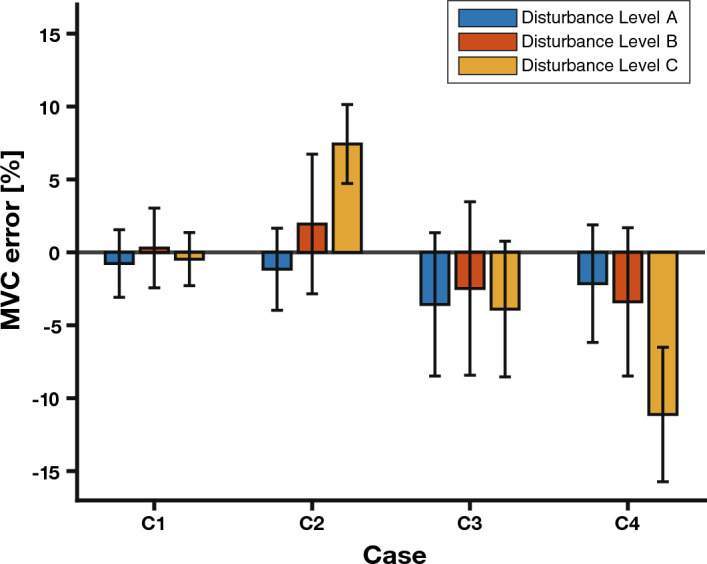
The bar and error bar graphs show the average MVC error and variance of the representative participants for each condition. The bar graph shows the average of each MVC error, and the error bar graph shows the variance of each MVC error.

Considering all the participant data, 95% of the power was concentrated below 2.6996 Hz on average. This finding proves that the effective cut-off frequency of the muscle is approximately 2–3 Hz and can be said to be a very physiologically valid value^5^. A third-order Butterworth filter was selected with a cut-off frequency of 6 Hz, which is more than twice that of the filter that was implemented using the filtfilt function of the MATLAB R2021b (The MathWorks, Inc.) library to preserve the original signal by the Nyquist theorem, (Fig. 4B). The MVC signal of the participant was filtered using the corresponding filter, and the MVC error was calculated based on the difference from the target MVC. In addition, the average and variance of the participants’ MVC errors were calculated for each condition (Fig. 5).

We studied the parameters predominantly involved in MVC maintenance in humans. We calculated the participants’ MVC errors quantitatively for each condition and analyzed the errors using a three-way repeated-measures ANOVA test for visual feedback (with two levels: i.e., providing visual feedback, VFB On; not providing visual feedback, VFB Off), target MVC (with two levels: 20% and 50%), and external disturbance (with three levels: external disturbance = 0, Disturbance Level A increasing external disturbance, Disturbance Level B; maximum external disturbance, Disturbance Level C) factors. Statistical analyses were performed using the IBM SPSS Statistics version 26 (IBM Corp.).

### Supplementary Information

Supplementary Information.

## Data Availability

Data are fully available through the corresponding author upon reasonable request.
