# Supplementary material for: Analysis of maintaining human maximal voluntary contraction control strategies through the power grip task in isometric contraction
Source: Sci Rep. 2024 Jan 12;14:1174. doi: 10.1038/s41598-023-51096-y (PMC10786847; doi:10.1038/s41598-023-51096-y)
Supplement: Supplementary file 1 — Supplementary Information. [file 41598_2023_51096_MOESM1_ESM.docx]

**S1 Table.** **Summary of statistical analysis of the MVC errors.**

| **Item** | **Variable** | **Test** | **Statistic** | **Confidence** |
| --- | --- | --- | --- | --- |
| A | The error of MVC under the conditions of visual feedback (VFB), each target MVC (MVC), each Disturbance Level (DL) | Three-way repeated measures  ANOVA | MVC error (VFB):  Mauchly's Test χ2(0) = 0.000,  p = nothing, ε = 1.000;  F (1,24) = 0.191;  MVC error (MVC):  Mauchly's Test χ2(0) = 0.000,  p = nothing, ε = 1.000;  F (1,24) = 51.492;  MVC error (DL):  Mauchly's Test χ2(2) = 7.067,  p = 0.029, ε = 0.791;  F (1.582,37.958) = 25.203;  MVC error (VFB * MVC):  Mauchly's Test χ2(0) = 0.000,  p = nothing, ε = 1.000;  F (1,24) = 15.685;  MVC error (VFB * DL):  Mauchly's Test χ2(2) = 13.053,  p = 0.001, ε = 0.698;  F (1.396,33.494) = 1.053;  MVC error (MVC * DL):  Mauchly's Test χ2(2) = 6.286,  p = 0.043, ε = 0.807;  F (1.614,38.736) = 3.056;  MVC error (VFB * MVC * DL):  Mauchly's Test χ2(2) = 8.270,  p = 0.016, ε = 0.768;  F (1.536,36.866) = 9.981; | MVC error: p = 0.666, partial η2 = 0.008,  power = 0.070,  corrected by Greenhouse-Geisser;  MVC error: p = 0.000, partial η2 = 0.682,  power = 1.000,  corrected by Greenhouse-Geisser;  MVC error: p = 0.000, partial η2 = 0.512,  power = 1.000,  corrected by Greenhouse-Geisser;  MVC error: p = 0.001, partial η2 = 0.395,  power = 0.967,  corrected by Greenhouse-Geisser;  MVC error: p = 0.337, partial η2 = 0.042,  power = 0.190,  corrected by Greenhouse-Geisser;  MVC error: p = 0.069, partial η2 = 0.113,  power = 0.502,  corrected by Greenhouse-Geisser;  MVC error: p = 0.001, partial η2 = 0.294,  power = 0.948,  corrected by Greenhouse-Geisser; |
| B | The error of MVC under the conditions of visual feedback | Bonferroni-corrected pairwise comparisons | Mean difference = 0.227,  Std. error = 0.518 | p = 0.666  lower bound = -0.843, upper bound = 1.296 |
| C | The error of MVC under the conditions of each target MVC | Bonferroni-corrected pairwise comparisons | Mean difference = 3.531,  Std. error = 0.492 | p < 0.0001  lower bound = 2.515, upper bound = 4.546 |
| D | The error of MVC under the conditions of each Disturbance Level (DL) | Bonferroni-corrected pairwise comparisons | DL A: DL B Mean difference = 2.897 Std. error = 0.336  DL A: DL C Mean difference = 0.599 Std. error = 0.525  DL B: DL C Mean difference = 2.298 Std.error = 0.411 | p < 0.0001  lower bound = 2.033, upper bound = 3.761  p = 0.796  lower bound = -0.752, upper bound = 1.950  p < 0.0001  lower bound = 1.242, upper bound = 3.355 |

**S2 Table.** **Summary of statistical analysis of the absolute MVC errors.**

| **Item** | **Variable** | **Test** | **Statistic** | **Confidence** |
| --- | --- | --- | --- | --- |
| A | The absolute error of MVC under the conditions of visual feedback (VFB), each target MVC (MVC), each Disturbance Level (DL) | Three-way repeated measures  ANOVA | MVC error (VFB):  Mauchly's Test χ2(0) = 0.000,  p = nothing, ε = 1.000;  F (1,24) = 62.443;  MVC error (MVC):  Mauchly's Test χ2(0) = 0.000,  p = nothing, ε = 1.000;  F (1,24) = 90.207;  MVC error (DL):  Mauchly's Test χ2(2) = 9.231,  p = 0.010, ε = 0.752;  F (1.503,36.074) = 18.449;  MVC error (VFB * MVC):  Mauchly's Test χ2(0) = 0.000,  p = nothing, ε = 1.000;  F (1,24) = 1.761;  MVC error (VFB * DL):  Mauchly's Test χ2(2) = 11.854,  p = 0.003, ε = 0.713;  F (1.426,34.219) = 55.190;  MVC error (MVC * DL):  Mauchly's Test χ2(2) = 5.403,  p = 0.067, ε = 0.827;  F (1.654,39.690) = 3.707;  MVC error (VFB * MVC * DL):  Mauchly's Test χ2(2) = 20.749,  p = 0.000, ε = 0.627;  F (1.254,30.107) = 3.989; | MVC error: p = 0.000, partial η2 = 0.722,  corrected by Greenhouse-Geisser;  MVC error: p = 0.000, partial η2 = 0.790,  corrected by Greenhouse-Geisser;  MVC error: p = 0.000, partial η2 = 0.665,  corrected by Greenhouse-Geisser;  MVC error: p = 0.197, partial η2 = 0.068,  corrected by Greenhouse-Geisser;  MVC error: p = 0.000, partial η2 = 0.849,  corrected by Greenhouse-Geisser;  MVC error: p = 0.041, partial η2 = 0.287,  corrected by Greenhouse-Geisser;  MVC error: p = 0.046, partial η2 = 0.160,  corrected by Greenhouse-Geisser; |
